# Family alcohol use, rather than childhood trauma, is more likely to cause male alcohol use disorder: findings from a case-control study in northern China

**DOI:** 10.1186/s12888-021-03566-8

**Published:** 2021-11-10

**Authors:** Xu Chen, Yunmeng Pan, Peiru Xu, Yi Huang, Nan Li, Yun Song

**Affiliations:** 1 Department of Substance Abuse, Shandong Mental Health Center Affiliated Shandong University, Shandong Mental Health Center Affiliated Jining Medical University, Jinan, China; 2 Office of academic studies, Jinan Science and Technology School, Jinan, China; 3 College of Mental Health, Jining Medical University, Jining, China; 4 School of Humanities and Social Sciences, Binzhou Medical University, Yantai, China; 5 Department of Neurology & Psychology, The First Affiliated Hospital of Shandong First Medical University & Shandong Provincial Qianfoshan Hospital, Shandong Institute of Neuroimmunology, Shandong Key Laboratory of Rheumatic Disease and Translational Medicine, Jinan, China

**Keywords:** Alcohol use disorder, Childhood, Trauma, Parents

## Abstract

**Objective:**

To explore the influence of childhood trauma and family alcohol use on male alcohol use disorder.

**Methods:**

We conducted a case-control study using Childhood Trauma Questionnaire (CTQ) and a structured interview involving 129 men with alcohol use disorder and 129 healthy male volunteers. The two groups were compared in terms of childhood trauma, parental drinking behavior, and attitudes toward childhood drinking.

**Results:**

Patients showed higher scores of CTQ than controls on childhood trauma experiences, including on the subscales of physical abuse, emotional abuse, sexual abuse, and emotional neglect. Higher proportions of patients than controls had fathers who drank seven or more times a week, and had mothers who were opposed to childhood drinking. Conversely, a smaller proportion of patients than controls had fathers who opposed childhood drinking. Patients were more likely than controls to have been induced to drink as children. Logistic regression analysis identified three risk factors for alcohol use disorder: induced drinking during childhood [odds ratio (OR) 6.09, 95% confidence interval (CI) 2.56–14.51], the father’s weekly alcohol consumption during the respondent’s childhood (OR 4.40, 95%CI 2.94–6.58) and history of smoking (OR 3.39, 95%CI 1.48–7.77). Conversely, more years of education were a protective factor against alcohol use disorder (OR 0.88, 95% CI 0.78–0.99).

**Conclusions:**

Men whose fathers drank frequently during their childhood and were encouraged to drink may be at increased risk of alcohol use disorder in adulthood. In fact these factors of family alcohol use appear to increase risk of alcohol use disorder among adult men more than exposure to childhood trauma does.

## Background

The etiology of alcohol use disorder (AUD) is complex and results from a combination of biological factors as well as psychological and sociological factors. Among them, biological factors cover many aspects such as genes, individual metabolism, central reward system, and changes in neurotransmitters such as dopamine and γ-aminobutyric acid [1, 2]. Psychological and sociological factors include personality traits, childhood experiences, social culture, and many other aspects. In terms of personality traits, such as low levels of empathy [3], high novelty seeking [4], high levels of impulsivity [5], depressive and anxious temperament [6], as typical personality characteristics of AUD, not only increase the risk of AUD, but also have impacts on emotional state, craving, and relapsing. In terms of childhood experiences, childhood trauma has received a lot of attention as an important and relatively independent factor. The World Health Organization (WHO) defines childhood trauma as the actual or potential harm done by a person who is obligated to raise, supervise, or care for a child [7]. These harms can include physical, emotional, and sexual abuse; neglect; and deprivation. All such harms are sufficient to compromise children’s health, survival, growth, development, and dignity.

A study in China found that rates of emotional, physical, and sexual abuse as well as emotional neglect during childhood are higher among patients with alcohol dependence than in the general population [8]. Other studies found that childhood traumatic experiences are an important risk factor for alcohol use disorders [9–11]. Studies by WHO and in eight Eastern European countries also found that emotional neglect in childhood trauma is significantly related to adolescent health-related risk behaviors such as alcohol abuse [12]. Alcohol abuse is 6–12 times more common among patients who suffered physical abuse in childhood than among those who did not [13]. Childhood trauma may lead to alcohol use disorder in adulthood through mechanisms based on post-traumatic stress or with the goal of “self-medication” [14–17]. Under the influence of physical and mental post-traumatic symptoms, patients may become socially maladjusted, coping unproductively with negative events and suffering distortions in cognition and personality. This causes various problems for patients, who resort to psychoactive substances such as alcohol for short-term self-healing and relief of anxiety and depression [18–21].

The family is the earliest and most important social environment for children’s growth experiences. The behavior of parents is commonly imitated by children [22]. Parental alcohol use may be a behavior imitated by the child. The parent’s attitudes and behaviors toward alcohol use may also be imitated by the child, which may play an important role in the development of alcohol use disorder. Therefore, how parents use alcohol may play a vital role in determining how children and adults use it, in addition to genetic factors [23]. The severity of parental alcohol use disorder affects the risk of alcohol use disorder in children, but how specific alcohol-related parental behaviors as well as other social and psychological factors may influence children’s behaviors toward alcohol has not been fully evaluated. Also unknown is whether these social and psychological factors are more important contributors to alcohol use disorder than childhood trauma. Many studies have found that parental alcohol use is a risk factor for childhood trauma [24–26], but whether parental alcohol-related behaviors and attitudes in themselves can affect children’s drinking behavior is unclear.

Therefore, we conducted a study to evaluate and compare the impacts of family alcohol use and childhood traumatic experiences on the development of alcohol use disorder among Han Chinese men from northern China.

## Methods

### Study population and study design

We conducted a case-control study including 129 men diagnosed with alcohol use disorder who were admitted to the Shandong Mental Health Center (Shandong, China) from January to December 2017. The study was approved by the Ethics Committee of Shandong Mental Health Center (2016R08).

Men were enrolled if they provided written informed consent, were 18–60 years old, had been diagnosed with “alcohol use disorder” based on the Fourth Edition of the Diagnostic and Statistical Manual of Mental Disorders (DSM-IV), had been hospitalized for at least two weeks or until severe physical withdrawal symptoms disappeared, and were able to complete the test and evaluation. Patients were excluded if they had major physical diseases or other mental disorders, including other addiction diseases, according to DSM-IV.

An equal number of healthy men from the general population were recruited from surrounding residential areas. To be enrolled, healthy controls had to be aged 18–60 years, score fewer than 7 points on the alcohol use disorders identification test (AUDIT) [27], have no major physical diseases or severe mental disorders, and provide written informed consent. Healthy volunteers were matched with patients according to age, such that age differences were less than 12 months.

To calculate minimal sample size, we drew from a 2005 epidemiological survey in Shandong Province, which indicated a prevalence of male alcohol use disorder of 11% [28]. A previous study in Shandong province found that prevalences of adverse childhood experiences were 42% among men with alcohol dependence and 16% in the general population [29]. Therefore, an odds ratio (OR) of 0.42/0.16 = 2.6 was predicted, which in order to detect with α = 0.05 and β = 0.2 would require at least 127 subjects in each group, as estimated using EpiCalc 2000 software.

### Questionnaire

A self-report questionnaire was designed to collect general demographic data of each subject, including age, nature of work (physical or non-physcial), marital status (unmarried, married, divorced, or widowed), years of education, smoking history (defined as smoking ≥10 cigarettes per day for at least one year at any time in the past), and family history of psychosis or alcohol addiction.

The Chinese version of AUDIT [27] was used to screen healthy volunteers before enrollment. The self-rating scale consists of 10 questions, the first eight of which provide five-level scores, and the last two provide three-level scores. Questions refer to alcohol consumption, drinking frequency, alcohol dependence, and forgetfulness. The Chinese version of the questionnaire was modified from the original English-language AUDIT [30] based on the Chinese context. The number of “standard cups” in questions 2 and 3 of the questionnaire was converted into the amount of liquor or beer with an alcohol content of 56%. The cut-off value was 7 points, and a score of at least 7 was defined as alcohol use disorder [27].

The Chinese version of the Childhood Trauma Questionnaire was used to evaluate the patients [31]. As a self-reported questionnaire, it has 28 items, divided into five categories: physical abuse, physical neglect, emotional abuse, emotional neglect, and sexual abuse. Each category has five items, and the remaining three items are for testing validity. Each item has a five-level score; therefore, the total score of the scale ranges from 25 to 125. The higher the score, the more serious the childhood trauma experience was.

### Interview content and process

A structured interview was conducted with all study subjects in a quiet room at Shandong Mental Health Center. Interviewees asked about the age at first drink, history of drunkenness and age at first drunkenness, the minimum number of drinks per week (a single drinking alcohol content of more than 20 g) by the father/mother during childhood (scoring: 0 = 0 times, 1 = 1–3 times, 2 = 4–6 times, 3 = 7 times or more), whether or not drinking was induced during childhood (those who had a clear memory of the induced drinking experience in childhood were counted as 1, otherwise 0), and father’s/mother’s attitude towards drinking (scoring: 0 = oppose, 1 = neutral, 2 = support).

### Statistical analysis

SPSS 26.0 (IBM, New York, NY, USA) was used for statistical analysis. Data were tested for normality. Skewed data were represented as the median (25th percentile, 75th percentile). The chi-squared test and Wilcoxon rank-sum statistics were used to compare the total scores on the Childhood Trauma Questionnaire scale, the scores for each factor, the drinking behavior of parents in childhood, and parents’ attitudes towards children’s drinking. Stepwise forward conditional logistic regression was used to evaluate the impact of various factors (Tables 1, 2, 3) on alcohol use disorder. Test levels were established at α = 0.05. Differences associated with *P* < 0.05 were considered significant.

**Table 1:** Baseline demographic characteristics and family history of the study population

Characteristic		Patients (*n* = 129)	Controls (*n* = 129)	z /χ^2^ (p)
Age (years)^a^		43.0 (33.0, 50.0)	43.00 (33.0, 50.0)	
Years of education^a^		9.00 (9.00, 12.0)	11.00 (9.00,12.0)	0.13 (0.90)
Nature of work, n (%)	physical	77 (29.84)	75 (29.07)	
	non-physical	52 (20.16)	54 (20.93)	0.13 (0.90)
History of smoking, n (%)	Yes	81 (31.40)	44 (17.05)	
	No	48 (18.60)	85 (32.95)	21.25 (< 0.01)
Marital status, n (%)	Single	3 (1.16)	8 (3.10)	1.23 (0.22)
	Married	107 (41.47)	106 (41.09)	0.00 (1.00)
	Divorced	17 (6.59)	14 (5.43)	0.38 (0.70)
	Widow	2 (0.78)	1 (0.39)	0.00 (1.00)
Family history of	Yes	8 (3.10)	5 (1.94)	
psychosis, n (%)	No	121 (46.90)	124 (48.06)	0.32 (0.57)
Family history of	Yes	11 (4.26)	4 (1.55)	
alcohol addiction, n (%)	No	118 (45.74)	125 (48.45)	2.55 (0.11)

^a^ Values are the median (25th percentile, 75th percentile)

**Table 2:** Comparison of category subscores and total scores on the Childhood Trauma Questionnaire between men with alcohol use disorder and paired healthy men

(Sub)score	Patients (*n* = 129)	Controls (*n* = 129)	z (p)
Physical abuse	5.00 (5.00, 7.00)	5.00 (5.00, 6.00)	2.94 (< 0.01)
Body neglect	8.00 (6.00, 12.00)	7.00 (5.00, 10.00)	2.97 (< 0.01)
Mental abuse	7.00 (5.00, 9.00)	6.00 (5.00, 7.00)	4.57 (< 0.01)
Mental neglect	8.00 (6.00, 9.00)	7.00 (5.00, 8.00)	3.52 (< 0.01)
Sexual abuse	5.00 (5.00, 5.00)	5.00 (5.00, 5.00)	2.66 (< 0.01)
Total score	34.00 (29.50, 40.00)	32.00 (29.00, 36.00)	4.77 (< 0.01)

**Table 3:** Comparison of household alcohol use between alcohol use disorder patients and healthy controls

Parameter	Patients (*n* = 129)	Controls (*n* = 129)	z/χ^2^(p)
Age at first drink, years^a^	17.0 (15.0, 19.0)	18.0 (16.0, 22.0)	3.88 (< 0.01)
Experienced drunkenness, n (%)	129 (50)	120 (46.51)	2.71 (< 0.01)
Age at first drunkenness, years^a^	19.0 (17.0,24.5)	21.0 (18.0, 27.0)	4.08 (< 0.01)
Minimum number of times the father drank alcohol per week during respondent’s childhood, n (%)			
0	5 (1.94)	62 (24.03)	7.95 (< 0.01)
1–3	15 (5.81)	51 (19.77)	4.99 (< 0.01)
4–6	8 (3.10)	6 (2.33)	0.27 (0.78)
7 or more	101 (39.15)	10 (3.88)	11.3 (< 0.01)
Minimum number of times the mother drank alcohol per week during respondent’s childhood, n (%)			
0	99 (38.37)	107 (41.47)	1.09 (0.27)
1–3	26 (10.08)	16 (6.20)	1.52 (0.13)
4–6	2 (0.78)	6 (2.33)	1.08 (0.28)
7 or more	2 (0.78)	0	0.71 (0.48)
Induced to drink during childhood, n (%)			
Ever	83 (32.17)	30 (11.63)	
Never	46 (17.83)	99 (38.37)	44.23 (< 0.01)
Father’s attitude towards childhood drinking, n (%)			
Oppose	34 (13.18)	56 (21.71)	2.74 (0.01)
Neutral	47 (18.22)	40 (15.50)	0.79 (0.43)
Support	48 (18.60)	33 (12.79)	1.88 (0.06)
Mother’s attitude towards childhood drinking, n (%)			
Oppose	98 (37.98)	42 (16.28)	6.87 (< 0.01)
Neutral	27 (10.47)	83 (32.17)	6.92 (< 0.01)
Support	4 (1.55)	4 (1.55)	0.36 (0.72)

^a^ values are medians (25th, 75th percentile)

## Results

### Baseline characteristics

In this study, 150 patients and 192 healthy volunteers were screened, and 130 matched pairs were included based on the inclusion criteria. After exclusion of one pair due to withdrawal of informed consent, 129 pairs of men with alcohol use disorders and age-matched healthy men were included in the final analysis (Table 1). There were no significant differences in the number of years of education, nature of work, marital status or family history between patients and controls. History of smoking was more frequent among patients.

### Comparison of childhood trauma experiences

Patients showed significantly higher total scores as well as category subscores on the Childhood Trauma Questionnaire than healthy subjects (Table 2).

Values are medians (25th percentile, 75th percentile).

### Comparison of family alcohol use

The age at first drink was significantly lower in patients than in controls, as was the age at first drunkenness (Table 3). A significantly higher proportion of patients had ever been drunk. The proportion of patients whose fathers did not drink alcohol or drunk only 1–3 times a week during childhood was significantly lower than the corresponding proportion of controls. Conversely, the proportion of patients whose fathers drank at least seven times a week during childhood was significantly higher than the corresponding proportion of controls, as was the proportion of patients who were induced to drink as children. There was no significant difference between the groups in the frequency of mothers’ drinking during the respondents’ childhood. Compared to controls, the proportion of patients whose fathers opposed childhood drinking was significantly lower, while the proportion whose mothers opposed childhood drinking was significantly higher. A lower proportion of patients had mothers with neutral attitudes towards childhood drinking.

### Multiple logistic regression to identify factors that influence alcohol use disorder

Conditional forward logistic regression to calculate likelihood ratios was performed with alcohol use disorder as the dependent variable and the factors in Tables 1, 2, 3 as independent variables (Table 4). (Marital status was assigned as a pseudo-variable before logistic regression.) The minimum number of times per week that the father consumed alcohol, whether the respondent was induced to drink during childhood, and history of smoking were identified as risk factors of alcohol use disorder. Conversely, years of education was a protective factor against alcohol use disorder.

**Table 4:** Logistic regression to identify childhood factors affecting alcohol use disorder among adult men

Variable	B	SE	χ^2^	p	OR	95% CI
Years of education	−0.15	0.07	5.26	0.03	0.86	0.75–0.98
Minimum number of times the father drank alcohol per week during childhood	1.48	0.21	51.80	< 0.01	4.40	2.94–6.58
Induced children to drink	1.81	0.44	16.67	< 0.01	6.09	2.56–14.51
History of smoking	1.22	0.42	8.38	< 0.01	3.39	1.48–7.77
Constant	−1.72	0.84	4.21	0.04	0.18	

Abbreviations: B, regression coefficients; CI, confidence interval; OR, odd ratio; SE, standard error

## Discussion

Through this study it was demonstrated that family alcohol use, which included parental drinking frequency, parental attitudes toward childhood drinking, being induced to drink as a child, age at first drink and at first drunkenness, may play a vital role in the occurrence of AUD. In fact, these factors may be stronger contributors to AUD than experience of trauma in childhood, education level or history of smoking.

In this study, we found that the ages at first drink and first drunkenness were significantly lower in Chinese men with alcohol use disorder than in healthy age-matched men. This may reflect the influence of early exposure to alcohol due to family alcohol use and childhood traumatic experiences. In particular, the father’s drinking frequency and whether he opposed drinking during the subjects’ childhood appeared to affect their risk of alcohol use disorder in adulthood. Premature exposure to drinking or even drunkenness may reflect parental neglect of children and tacit approval of drinking, which would also increase risk of alcohol use disorder.

Han Chinese in northern China are more traditional than other ethnic groups or than Han Chinese in the south of the country [32]. The status of men in their families is higher than that of females, and male drinking is more acceptable than in other ethnicities. This is closely related to the influence of traditional Chinese Confucianism and local cultural customs. Therefore, fathers’ drinking behavior and attitudes may influence children, especially sons, more strongly than mothers’ behavior and attitudes. Children’s understanding of how men “should” behave originates mostly with the father [33, 34]. Therefore, the more the father drinks per week, the more likely the son will develop an alcohol use disorder during adulthood. The proportion of healthy controls whose fathers opposed childhood alcohol consumption was higher than the proportion of patients, suggesting that fathers’ attitudes towards their children’s drinking is one of the factors that affect the risk of alcohol use disorder [35]. Many families in northern China often encourage their children to try drinking when they have dinner during major festivals, which is partly related to local customs and also often serves as a form of entertainment at the table. However, this behavior often lowers children’s vigilance toward alcohol use, and it promotes the perception that drinking can be an effective way to attract attention from relatives and friends. At the same time, the encouragement from relatives and friends serves as implicit consent to children’s drinking. In this way, the idea that drinking is a typical male behavior is solidified in children’s minds. In the present work, we found that induced drinking during childhood is one of the most important risk factors for alcohol use disorder among adult men. This suggests that, regardless of the purpose of induced drinking during childhood, it often leads to alcohol use disorder in adulthood. Indeed, a previous study in Australia found that parents’ providing alcohol to their children greatly increases the risk that the children will suffer alcohol use disorder and alcohol-related harm in adolescence [36].

We observed that the proportion of men with alcohol use disorder whose mothers opposed childhood alcohol consumption was much higher than the proportion of healthy men. This may be related to the higher frequency of fathers’ drinking among people with alcohol use disorder, which may elicit strong opposition from mothers as a way to protect their children. Discrepant attitudes between mother and father towards drinking in childhood may trigger cognitive conflict in the child about drinking. Boys may then tend to side with the “masculine” view, rendering them more vulnerable to alcohol use disorder [35].

Childhood trauma experiences were more frequent among our men with alcohol use disorder than among our healthy controls. Childhood traumatic experiences lead to brain dysfunction, which may involve changes in neuromorphology, neural pathways, and gene expression [37, 38]. Functional magnetic resonance imaging studies have shown that the activation of certain brain regions is more difficult in individuals who have experienced childhood trauma [39, 40], which can lead to abnormal functions in the central reward system [41]. Childhood traumatic experiences also directly trigger negative emotions such as self-denial, pessimism, and anxiety [42]. In order to avoid these negative emotions, individuals often resort to alcohol, tobacco, and other psychoactive substances, increasing the risk of alcohol use disorder.

Negative emotions arising from childhood trauma also lead to increased sensitivity and alertness to external stimuli. Excessive sensitivity and alertness can cause anxiety, impulsivity, and uncontrollable emotions, resulting in decreased self-control when using psychoactive substances. Moreover, the availability of alcohol makes drinking one of the most convenient and legal ways to relieve anxiety, reduce sensitivity and alertness, and feel less alone [42]. Indeed, abuse or neglect during childhood can lead to emotional and behavioral problems and increase the risk of mental illnesses during adulthood, including substance use disorders and schizophrenia [42–46],

In contrast to these various risk factors, we observed that higher education level was a protective factor against alcohol use disorder in our sample. This may be because a higher education level improves cognition and control of alcohol use, reducing risk of its abuse [47, 48]. In general, higher education level in children is associated with higher parental education level and income, and more affluent families usually show more self-control in using alcohol [49] and may protect their children more from trauma [50]. The fathers and mothers in more affluent households are also more likely to share similar attitudes towards childhood drinking [49]. In this way, higher education level may directly and indirectly decrease risk of alcohol use disorder.

We found that a history of smoking was much more frequent among men with alcohol use disorder than among healthy men. This may reflect that tobacco is one of the most common and easily available, legal psychoactive substances, often used in conjunction with alcohol. In addition, a family’s positive attitude towards smoking is often similar to the family’s positive attitude towards the use of alcohol [51–53]. Tobacco and alcohol use disorders may, in fact, be linked to similar genetic polymorphisms [54].

Our study presents several limitations, such as the small, mono-sex sample, sampling in northern China, and unmatched education level. Furthermore, we excluded comorbidity of other mental diseases, did not identify clearly the family history of mental illness or addiction diseases and did not take into account parenting styles or personality traits. Our work needs to be confirmed in a larger sample that takes into account a wider range of biological, social, and psychological factors. For example, parenting styles have been associated with alcohol-related problems in childhood [55], and personality traits can influence risk of alcohol use disorder [56].

Despite these limitations, our study provides evidence that family alcohol use may contribute more strongly than childhood trauma to alcohol use disorder in adult men. Our results may help guide family-based policies for prevention of alcohol use disorder.

## Data Availability

The datasets used and/or analyzed during the current study are available from the corresponding author on reasonable request.
